# Blood Flow Restriction Exercise: Considerations of Methodology, Application, and Safety

**DOI:** 10.3389/fphys.2019.00533

**Published:** 2019-05-15

**Authors:** Stephen D. Patterson, Luke Hughes, Stuart Warmington, Jamie Burr, Brendan R. Scott, Johnny Owens, Takashi Abe, Jakob L. Nielsen, Cleiton Augusto Libardi, Gilberto Laurentino, Gabriel Rodrigues Neto, Christopher Brandner, Juan Martin-Hernandez, Jeremy Loenneke

**Affiliations:** ^1^Faculty of Sport, Health and Applied Sciences, St Marys University, London, United Kingdom; ^2^School of Exercise and Nutrition Sciences, Institute for Physical Activity and Nutrition, Deakin University, Geelong, VIC, Australia; ^3^Department of Human Health and Nutritional Science, University of Guelph, Guelph, ON, Canada; ^4^Murdoch Applied Sports Science Laboratory, Discipline of Exercise Science, Murdoch University, Perth, WA, Australia; ^5^Owens Recovery Science, San Antonio, TX, United States; ^6^Department of Health, Exercise Science, and Recreation Management, Kevser Ermin Applied Physiology Laboratory, University of Mississippi, Oxford, MS, United States; ^7^Department of Sports Science and Clinical Biomechanics, University of Southern Denmark, Odense, Denmark; ^8^MUSCULAB – Laboratory of Neuromuscular Adaptations to Resistance Training, Federal University of São Carlos (UFSCar), São Carlos, Brazil; ^9^School of Physical Education and Sport, University of São Paulo, São Paulo, Brazil; ^10^Coordination of Physical Education/Professional Master’s in Family Health, Nursing and Medical Schools, Nova Esperança (FAMENE/FACENE), João Pessoa, Brazil; ^11^Sport Science Department, Aspire Academy for Sports Excellence, Doha, Qatar; ^12^I+HeALTH Research Group, Department of Health Sciences, Faculty of Health Sciences, Miguel de Cervantes European University, Valladolid, Spain

**Keywords:** blood flow restriction exercise, kaatsu training, occlusion training, BFR exercise, resistance training

## Abstract

The current manuscript sets out a series of guidelines for blood flow restriction exercise, focusing on the methodology, application and safety of this mode of training. With the emergence of this technique and the wide variety of applications within the literature, the aim of this review is to set out a current research informed guide to blood flow restriction training to practitioners. This covers the use of blood flow restriction to enhance muscular strength and hypertrophy via training with resistance and aerobic exercise and preventing muscle atrophy using the technique passively. The authorship team for this article was selected from the researchers focused in blood flow restriction training research with expertise in exercise science, strength and conditioning and sports medicine.

## Introduction

Blood flow restriction (BFR) is a training method partially restricting arterial inflow and fully restricting venous outflow in working musculature during exercise (Scott et al., 2015). Performing exercise with reduced blood flow achieved by restriction of the vasculature proximal to the muscle dates back to Dr. Yoshiaki Sato in Japan, where it was known as “kaatsu training,” meaning “training with added pressure.” Kaatsu training is now performed all over the world and is more commonly referred to as “BFR training" and achieved using a pneumatic tourniquet system (Wernbom et al., 2008; Loenneke et al., 2012d).

The technique of BFR in the muscle using a pneumatic tourniquet system involves applying an external pressure, typically using a tourniquet cuff, to the most proximal region of the upper and/or lower limbs. When the cuff is inflated, there is gradual mechanical compression of the vasculature underneath the cuff, resulting in partial restriction of arterial blood flow to structures distal to the cuff, but which more severely affects venous outflow from under the cuff that is proposed to also impede venous return. Compression of the vasculature proximal to the skeletal muscle results in inadequate oxygen supply (hypoxia) within the muscle tissue (Manini and Clark, 2009; Larkin et al., 2012). Furthermore, the diminution of venous blood flow results in blood pooling within the capillaries of the occluded limbs, often reflected by visible erythema. The level of blood pooling may be influenced by the amount of pressure applied. In addition to this, when muscular contractions are performed under conditions of BFR, there is an increase in intramuscular pressure beneath the cuff (Kacin et al., 2015), which further disturbs blood flow.

Whilst the number of research groups and studies investigating BFR have grown, so too has the number of practitioners using this mode of training (Patterson et al., 2017). This is positive, however, evidence from Patterson et al. (2017) suggests that practitioners are unclear on how to use and apply BFR in line with current research informed standards. For example, there was a wide range of pressures applied by practitioners that resulted in unintended consequences such as a large incidence of numbness following BFR. Therefore, the aim of this review is to provide a current, research informed guide to BFR from a group of world leading experts in the field. It is envisaged that this will facilitate practitioners to be more informed and clearer in deciding the reasons why they should apply BFR, how they should apply BFR as well as understanding the safety issues associated with this BFR training. For a more detailed understanding of the mechanisms of BFR exercise we refer readers to the following review articles (Wernbom et al., 2008; Pearson and Hussain, 2015).

## Application of BFR

BFR is applied during both voluntary resistance exercise (BFR-RE) and aerobic exercise (BFR-AE), and also passively without exercise (P-BFR). More recent research has examined the combination of BFR with non-traditional exercise modalities, such as whole-body vibration techniques and neuromuscular electrical stimulation.

### Protocols for Enhanced Muscle Strength and Hypertrophy

In the following section, an overview of the BFR literature aiming at increasing maximal skeletal muscle strength and muscle mass will be provided. Tables 1, 2 provide an overview of the recommendations for the application of BFR-RE and BFR-AE, respectively.

**Table 1:** Model of exercise prescription with BFR-RE.

	Guidelines
Frequency	2–3 times a week (>3 weeks) or 1–2 times per day (1–3 weeks)
Load	20–40% 1RM
Restriction time	5–10 min per exercise (reperfusion between exercises)
Type	Small and large muscle groups (arms and legs/uni or bilateral)
Sets	2–4
Cuff	5 (small), 10 or 12 (medium), 17 or 18 cm (large)
Repetitions Pressure	(75 reps) – 30 × 15 × 15 × 15, or sets to failure 40–80% AOP
Rest between sets	30–60 s
Restriction form	Continuous or intermittent
Execution speed	1–2 s (concentric and eccentric)
Execution	Until concentric failure or when planned rep scheme is completed


**Table 2:** Model of exercise prescription with BFR-AE.

	Guidelines
Frequency	2–3 times a week (>3 weeks) or 1–2 times per day (1–3 weeks)
Intensity	<50% VO_2_ max or HRR
Restriction time	5–20 min per exercise
Type	Small and large muscle groups (arms and legs / uni or bilateral)
Sets Pressure	Continuous or intervals 40–80% AOP
Cuff	5 cm (small), 10 or 12 cm (medium), 17 or 18 cm (large)
Exercise mode	Cycling or walking


### BFR-RE

Increases in muscle hypertrophy and strength with BFR-RE are extensively documented. In recent years, a number of systematic reviews and meta-analyses have demonstrated BFR-RE to effectively increase skeletal muscle strength and/or hypertrophy in healthy young (Loenneke et al., 2012d; Slysz et al., 2016; Lixandrão et al., 2018) and older (Centner et al., 2018a; Lixandrão et al., 2018) populations, as well as load compromised populations in need of rehabilitation (Hughes et al., 2017). Various measures of muscle strength have been shown to improve in response to BFR-RE interventions, including dynamic isotonic (Burgomaster et al., 2003; Moore et al., 2004), isometric (Takarada et al., 2000a; Moore et al., 2004) and isokinetic strength (Takarada et al., 2000c, 2004 Burgomaster et al., 2003; Moore et al., 2004), as well as rate of force development/explosive strength capacity (Nielsen et al., 2017b). It is well documented that muscle hypertrophy and strength adaptations with BFR-RE are significantly greater than those achieved with low-load resistance exercise (LL-RE) alone in most (Takarada et al., 2002, 2004; Abe et al., 2005a,b,c; Yasuda et al., 2005) but not all studies (Farup et al., 2015). Such adaptations have been observed after only 1–3 weeks (Abe et al., 2005a,b, 2006; Fujita et al., 2008; Nielsen et al., 2012; Yasuda et al., 2005). These timescales for early increases in strength are mirrored in high-load resistance exercise (HL-RE) research (Blazevich et al., 2017), however, this is not typically the case for muscle mass where adaptations are not usually observed in 1–3 weeks following HL-RE (Damas et al., 2016).

Although increases in muscle size may be partly a result of the acute edema observed during and after BFR-RE (Loenneke et al., 2012c; Pearson and Hussain, 2015), improvements are still observed between 2 and 10 days post-training (Abe et al., 2005a; Fujita et al., 2008; Nielsen et al., 2012). Thus, it appears that BFR-RE allows for early addition of skeletal muscle mass; it should be noted though that this early muscle growth is likely due to the ability to use BFR-RE with a high training frequency, which is not always possible with HL-RE. For example, the lower loads used during BFR-RE to not take as long to recover from as HL-RE and thus due to these lower mechanical demands this may allow for a higher training frequency. Muscle hypertrophy with conventional training frequency (2–3 times per week) has been observed following longer training durations of 3 weeks (Ladlow et al., 2018), 5 weeks (Manimmanakorn et al., 2013), 6 weeks (Thiebaud et al., 2013), and ≥8 weeks of training (Moore et al., 2004; Libardi et al., 2015; Yasuda et al., 2016; Cook et al., 2017). BFR-RE improves muscle strength in comparison to LL-RE alone (Hughes et al., 2017) but generally show less gain in muscle strength compared to HL-RE (Loenneke et al., 2012d; Hughes et al., 2017; Lixandrão et al., 2018). Recent meta-analysis of Lixandrão et al. (2018) showed superior muscle strength gains for HL-RE as compared with BFR-RE, even when adjusting for potential moderators [e.g., test specificity (dynamic or isometric), cuff width, absolute occlusion pressure and occlusion pressure prescription method]. On the other hand, the same meta-analysis showed that BFR-RE induces comparable increases in muscle mass when compared to HL-RE, regardless of cuff width, absolute occlusion pressure and occlusion pressure prescription method. Thus, we suggest that although the muscle strength gains observed in BFR-RE are lower compared to HL-RE, the BFR is more effective than LL-RE alone and can be used when HL-RE is not advisable (e.g., post-operative rehabilitation, cardiac rehabilitation, inflammatory diseases, and frail elderly). When considering muscle mass growth, both BFR-RE and HL-RE seem equally effective. Disuse atrophy is a frequent complication in clinical populations making BFR-RE a potential alternative to HL-RE specifically for muscle mass loss.

### Determining Cuff Pressure

The amount of pressure required to cease blood flow to a limb [i.e., arterial occlusion pressure (AOP)] is related to a range of individual limb characteristics; tourniquet shape, width and length, the size of the limb or an individual’s blood pressure (Loenneke et al., 2012b, 2015; Jessee et al., 2016; McEwen et al., 2018). A bigger limb will require a greater cuff pressure to fully restrict arterial blood flow, and this is true across a range of cuff widths (Loenneke et al., 2012b). Some researchers have suggested that the pressure could be set relative to the individual, cuff width, and cuff material by setting the pressure relative to the arterial occlusion pressure of the cuff that will be utilized during exercise (% AOP; Patterson et al., 2017; McEwen et al., 2018). This can be done by inflating the cuff being used during exercise up to the point where blood flow ceases (100% AOP) and using a percentage of that pressure (e.g., 40–80% of AOP) during exercise. Although some have applied pressures relative to brachial systolic blood pressure (SbP) (traditional blood pressure; Brandner et al., 2015), this may not provide a consistent reduction in blood flow unless the cuff used for traditional blood pressure is the same cuff used during exercise (Loenneke et al., 2012b). How well traditional blood pressure in the arm applies to a leg (larger limb) is also something to consider with this method (Loenneke et al., 2016). Additionally, SbP has been found to correlate poorly with measurements of arterial occlusion pressure (Younger et al., 2004). Despite some researchers recommending the pressure be made relative to the exercised limb, the majority of early studies applied the same absolute pressure to each individual, independent of cuff width and limb size. These pressures have ranged from absolute pressures as low as 50 mmHg (Kubota et al., 2011) to as high as 300 mmHg (Cook et al., 2007). Although the majority of studies have produced beneficial muscular adaptations with the same absolute pressures applied to each individual, it appears that greater BFR pressure can augment the cardiovascular response and often induces discomfort associated with this type of exercise (Jessee et al., 2017; Mattocks et al., 2017). It is therefore recommended to set pressure during BFR exercise based on measurement of AOP, with pressures ranging from 40 to 80% of AOP having evidence to support their efficacy.

### Cuff Width

The amount of pressure required to cease blood flow to a limb (i.e., AOP) is largely determined by the width of the cuff being applied to the limb; a wider cuff requiring a lower pressure (Crenshaw et al., 1988; Loenneke et al., 2012b; Jessee et al., 2016), essentially due to the greater surface area to which pressure has been applied. This is an important point as there are a wide range of cuff widths (3–18 cm) used in the BFR literature and setting two differently sized cuffs to the same pressure may produce a completely different degree of limb BFR (Rossow et al., 2012). It is noted that applying a relative pressure of 40% AOP does not result in a 40% reduction in blood flow (Mouser et al., 2017b). Nevertheless, a recent study found that applying pressure as a % of AOP to three different sized cuffs produce a similar change in resting blood flow (Mouser et al., 2017a). This study found that a wider cuff required less absolute pressure to restrict blood flow at any given % of AOP, but that a narrow cuff inflated to a higher absolute pressure (but same % of AOP as wide cuff) had a similar reduction in blood flow. Although lower pressures can be used with a wider cuff, this does not necessarily equate to a safer stimulus but reflects each cuff size’s inherent ability to apply pressure through layers of tissue within a limb (Crenshaw et al., 1988). Lastly, we acknowledge that there may be some attenuation of growth directly under where the cuff is applied (Kacin and Strazar, 2011; Ellefsen et al., 2015), although one study suggests that this attenuation of growth may be prevented if a % of AOP is applied (Laurentino et al., 2016). Therefore, it is recommended that a wide variety of cuff widths can be used if pressure is set appropriately by using AOP. It should be noted that the wider the cuff the lower overall pressure will be needed, however, the use of extremely wide cuffs may limit movement during exercise.

### Cuff Material

Throughout the literature, both elastic and nylon cuffs are commonly utilized. In the lower body (Loenneke et al., 2013, 2014b), there appears to be little difference in resting arterial occlusion or repetitions to concentric failure (surrogate for blood flow) using cuffs of the same width but made of two different materials (elastic vs. nylon). In the upper body (Buckner et al., 2017), using cuffs of different material but similar size (3 vs.5 cm), there were large differences in resting AOP that seem unlikely explained by the slight difference in cuff width. However, when the pressure was made as a % of AOP to each cuff, the repetitions to volitional failure were similar between the two different cuff materials. This provides some evidence that the reduction of blood flow during the exercise was likely similar between cuff sizes. It appears that any difference in cuff material could be corrected for by simply applying a pressure relative to the total AOP specific for each cuff. Although studies have never directly compared cuff materials over the course of a training study, there is no available evidence to suggest that one cuff material would be superior to another. Further, both elastic and nylon cuffs have been utilized in the literature and have shown beneficial muscular adaptations (Fahs et al., 2015; Kim et al., 2017). Considering these collective findings together, the material of the cuff does not appear to impact the outcomes of BFR-RE.

### Exercise Load, Volume, Rest Periods, Duration, and Frequency

#### Exercise Load

The pressure applied during exercise may also be dictated to some degree by the relative load lifted during resistance exercise. For the majority of individuals exercising with loads corresponding to 20–40% of an individual’s maximum strength level (e.g., 1-RM) will likely maximize muscle growth and strength (Lixandrao et al., 2015; Counts et al., 2016). When loads used are at the bottom end of this recommendation (e.g., ∼20% of 1-RM), a higher pressure (∼80% AOP) may be required necessary to elicit muscle growth (Lixandrao et al., 2015), however, further study is warranted to confirm this. The majority of studies have investigated the elbow flexors and knee extensors and it is unknown whether different muscle groups require different pressure recommendations. For example, it has been suggested that targeting muscle groups proximal to the cuff may require a higher applied pressure for maximal adaptation (Dankel et al., 2016). In conclusion, we suggest that exercise loads between 20 and 40% 1RM be used because this range of loads has consistently produced muscle adaptations when combined with BFR.

#### Volume

In the BFR-RE literature, a common and frequently used set and repetition scheme exists that involves 75 repetitions across four sets of exercises, with 30 repetitions in the first set and 15 repetitions in each subsequent set (Yasuda et al., 2006, 2010a,b, 2011a,b, 2012; Madarame et al., 2008; Rossow et al., 2012; Ozaki et al., 2013; Loenneke et al., 2016; May et al., 2017). It is also common to complete 3–5 sets to concentric failure during BFR-RE (Takarada et al., 2002; Cook et al., 2007, 2013; Loenneke et al., 2012a; Manini et al., 2012; Nielsen et al., 2012; Ogasawara et al., 2013; Fahs et al., 2015). Furthermore, repetitions to failure may not be needed in practical settings, such as post-surgery rehabilitation of clinical populations. For example, doubling this volume of load lifted does not appear to augment any adaptations (Loenneke et al., 2011b; Martín-Hernández et al., 2013), although the dose-response relationship between volume and adaptation still needs further clarity. Therefore, it is suggested 75 repetitions, across four sets (30, 15, 15, 15) is sufficient volume to lead to adaptations in most people. Working to failure is another possibility to induce adaptations but may not always be required.

#### Rest Periods

Inter-set rest periods used during BFR-RE are generally short and typically the restriction is maintained throughout this period. For example, Loenneke et al. (2012d) conducted a meta-analysis that demonstrated strength adaptations with both 30 and 60 s inter-set rest periods. Some acute research has used rest periods as long as 150 s (Loenneke et al., 2010), but this was not found to increase metabolic stress any more than LL-RE, and thus may not provide training benefits. However, rest periods of both 30 s (Yasuda et al., 2010a, 2015b; Loenneke et al., 2011a) and 30–60 s (Madarame et al., 2010; Patterson and Ferguson, 2010, 2011; Yasuda et al., 2015b; Loenneke et al., 2016; Ladlow et al., 2018) are common within the BFR literature, which reflects the recommendations for achieving skeletal muscle hypertrophy (Kraemer and Ratamess, 2004). On occasions it is not always required to maintain pressure during rest periods. For example, Yasuda et al. (2013) demonstrated similar muscle activation with both continuous and intermittent pressure during rest periods, but only when a high cuff pressure was applied. Overall we recommend rest periods should constitute 30–60 s, however, intermittent BFR may reduce swelling/metabolic stress compared with continuous, which could limit the stress for adaptation.

#### Frequency

Traditionally, it is recommended to perform resistance training 2–4 times per week to stimulate skeletal muscle hypertrophy and strength adaptations (Fleck and Kraemer, 2004; Kraemer and Ratamess, 2004). Increases in muscle hypertrophy and strength have been reported with BFR-RE twice weekly (Takarada et al., 2000b, 2002; Laurentino et al., 2008; Madarame et al., 2008), with a recent review advocating that 2–3 BFR-RE sessions per week with progressive overload is sufficient for enhanced strength and hypertrophy adaptations (Scott et al., 2015). Some BFR research has implemented training twice daily (Abe et al., 2005b; Yasuda et al., 2005, 2010b; Nielsen et al., 2012), which may be used to accelerate recovery in a clinical rehabilitation setting (Ohta et al., 2003; Ladlow et al., 2018). In conclusion, high frequency approaches (1–2 times per day) may be used for short periods of time (1–3 weeks), however, under periods of normal programming, 2–3 sessions per week are ideal.

#### Duration of Training Programmes

Regarding duration of BFR-RE programmes, muscle hypertrophy and strength adaptations have been observed in short time frames, such as 1–3 weeks (Abe et al., 2005b,c; Yasuda et al., 2005; Fujita et al., 2008; Nielsen et al., 2012). Most studies have examined muscle hypertrophy and strength adaptations over time frames >3 weeks duration (Burgomaster et al., 2003; Moore et al., 2004; Abe et al., 2006; Iida et al., 2011; Nielsen et al., 2012; Yasuda et al., 2012; Martín-Hernández et al., 2013; Luebbers et al., 2014; Kang et al., 2015).

### BFR-AE

BFR-AE has been systematically reviewed (including a meta-analysis) demonstrating the effectiveness of increased strength and hypertrophy in young (Slysz et al., 2016) and older populations (Centner et al., 2018a). The application of BFR-AE usually occurs during either walking (Abe et al., 2006) or cycling exercise (Abe et al., 2010a; Conceição et al., 2019). Adaptations for strength and skeletal muscle hypertrophy have been demonstrated as early as 3 weeks (Abe et al., 2006) but most effective after at least 6 weeks of training (Slysz et al., 2016). Skeletal muscle strength has been shown to increase by 7–27% (Abe et al., 2006, 2010a,b; Ozaki et al., 2011a,b; de Oliveira et al., 2016; Clarkson et al., 2017a; Conceição et al., 2019) and hypertrophy by 3–7% (Abe et al., 2006, 2010a,b; Ozaki et al., 2011a,b; Sakamaki et al., 2011; Conceição et al., 2019) following BFR-AE. Furthermore, this mode of exercise also improves functional ability in a range of tasks (Clarkson et al., 2017a), demonstrating the impact of increased strength and muscle mass from BFR-AE on activities relevant to daily living, health and wellbeing. Alongside these changes BFR-AE can also lead to significant improvements in aerobic capacity across young (Slysz et al., 2016), old (Abe et al., 2010a), and even trained individuals (Park et al., 2010) but this is not always the case. The intensities used during BFR-AE are generally low in nature (45% heart rate reserve or 40% VO_2_ max; Abe et al., 2010a; Clarkson et al., 2017a; Conceição et al., 2019), and in some cases have not been standardized (Abe et al., 2006, 2010b; Clarkson et al., 2017a) or have been implemented with a wide variety of cuff widths and pressures. A smaller body of literature has examined a variation on BFR-AE, wherein the BFR is applied immediately after the aerobic effort. Adaptations reveal an exaggerated improvement in VO_2_max, and the potential for greater aerobic adaptations as a result of an acute upregulation of protein signaling (Taylor et al., 2016), as has also been shown in highly trained athletes comparing BFR-AE with matched systemic hypoxia (Christiansen et al., 2018). Unlike BFR-RE there has been a lack of standardization of pressure during BFR-AE which should be a focus in the future to optimize responses and gain greater understanding of the muscle adaptations to training with BFR-AE.

### Protocols for Prevention of Strength Loss and Atrophy

In the following section, an overview over the BFR literature aiming at reducing the loss of skeletal muscle strength and muscle mass will be provided.

### P-BFR

Another strategy for the use of BFR involves applying the cuffs to limbs without undertaking exercise (i.e., P-BFR). Although these approaches have not received substantial research attention, available data indicates that intermittent application of P-BFR may offset muscle atrophy and strength loss during periods of bed rest or immobilization (Takarada et al., 2000b; Kubota et al., 2008, 2011). This could theoretically provide benefits for patients following orthopedic surgeries such as anterior cruciate ligament (ACL) reconstruction and total knee arthroplasty, as less muscle mass and strength need to be regained in the rehabilitation phase. P-BFR is a similar technique to that performed during ischemic preconditioning, namely periods of ischemia followed by periods of reperfusion. To date this approach has been used to attenuate the decline in muscle mass and strength following ACL surgery (Takarada et al., 2000b), during cast immobilization (Kubota et al., 2008, 2011), and in patients in intensive care (Barbalho et al., 2018). Furthermore, P-BFR has been shown to elicit enhanced local skeletal muscle oxidative capacity and cardiovascular improvements such as increased endothelial–dependent vasodilation and vascular conductance (∼14%) in as little as 7 days (Jones et al., 2014; Jeffries et al., 2018). Similar observations have also been made following intermittent exposure over 4 weeks (Kimura et al., 2007) and 8 weeks (Jones et al., 2015).

To date, P-BFR has been implemented following a standard protocol that was originally developed by Takarada et al. (2000b). This protocol consists of 5 min of restriction followed by 3 min of reperfusion applied for 3–4 sets. Researchers have so far implemented this P-BFR once or twice per day and for a duration of 1–8 weeks (Takarada et al., 2000b; Kubota et al., 2008, 2011; Jones et al., 2014, 2015; Jeffries et al., 2018). It should be acknowledged though that studies have not yet investigated other protocols using different durations of BFR and reperfusion or altering the ratio of time spent with the cuff inflated vs. deflated. The pressures used during P-BFR have varied from 50 mmHg (Kubota et al., 2011) to 260 mmHg in some participants (Takarada et al., 2000b). As it stands, there is no definitive pressure allocated in the literature investigating P-BFR, although it does appear that relatively high pressures may provide the most potent protective effects against disuse atrophy given the associated complete occlusion to flow (Takarada et al., 2000b; Kubota et al., 2008). Unlike BFR exercise, the use of AOP has not been prevalent in this section of the research. It is likely that the high pressures used in some research can completely occlude blood flow to and from the limb, but this is also dependent on other factors such as cuff and limb size (Loenneke et al., 2012b).

### BFR With Electrical Stimulation (BFR-ES)

Recently evidence has emerged for the use of BFR-ES. To date there is very little evidence in this area. Natsume et al. (2015) demonstrated increased muscle thickness and strength over a 2 weeks period in untrained male participants following twice per day BFR-ES. Intensity appears to have a dose-response relationship with muscular adaptation, with significant strength gains observed when a maximally tolerable stimulation intensity is used (Slysz and Burr, 2018), and positive associations between training intensity and increased in strength (*r* = 0.8) as well as cross-sectional area of both fast (*r* = 0.9) and slow (*r* = 0.7) fibers (Natsume et al., 2018). Furthermore, 6 weeks of unilateral low-intensity BFR-ES increased the CSA of the extensor carpi radialis longus by 17% more than ES alone in the contralateral arm of spinal cord injury patients (Gorgey et al., 2016). Alongside this, these patients also demonstrated improved vascular function, as evidenced by an increase in flow mediated dilatation (FMD). While BFR-ES is an interesting new avenue in this field, more research is needed before evidence-based recommendations for practitioners can be made.

### SAFETY

The following sections will cover the safety aspects that need to be considered when implementing BFR.

### Cardiovascular Response to BFR-RE

During exercise the rise of oxygen demand in the active skeletal muscles is matched by both central and peripheral vascular responses. Heart rate (HR) and stroke volume (SV) determine the total cardiac output (CO) that is distributed by vascular resistance (Hogan, 2009). Mechanisms regulating blood flow (BF) involve the central nervous system (i.e., modulation of sympathetic tone) and peripheral feedback arising from regional (i.e., venules and arterioles) and local mechanisms (i.e., the capillary beds) (Murrant and Sarelius, 2015). Local control of vasomotor tone is dependent upon metabolic, mechanical and endothelial factors. The integrated responses of increased metabolic stress, external compression of the arterial wall and shear stress of the endothelium limit autonomic sympathetic control of vasomotor tone eventually leading to a balanced level of vasodilation within active muscle that provides adequate distribution of CO (Saltin et al., 1998), and these factors are known to be affected by BFR-RE (Mouser et al., 2017a; Credeur et al., 2010). The uniqueness of BFR-RE arises from the externally applied pressure compressing blood vessels and the surrounding soft tissue that could mediate an altered cardiovascular response. Henceforth, evidences of the main central and peripheral short- and long-term vascular adaptations are presented.

### Central Vascular Response to BFR-RE

The effect of BFR-RE on the central cardiovascular response is dependent upon the level of BFR (Rossow et al., 2012), mode of exercise (i.e., BFR-RE vs. BFR-AE) (Staunton et al., 2015) and mode of application (i.e., continuous vs. intermittent BFR) (Brandner et al., 2015; Neto et al., 2016). BFR acutely affects central hemodynamic parameters when it is combined with RT (Takano et al., 2005; Rossow et al., 2011, 2012; Fahs et al., 2012; Vieira et al., 2013; Downs et al., 2014; Brandner et al., 2015; Staunton et al., 2015; Neto et al., 2016; Poton and Polito, 2016; Libardi et al., 2017; May et al., 2017), AE (Renzi et al., 2010; Kumagai et al., 2012; Sugawara et al., 2015; Staunton et al., 2015; May et al., 2017) or even in the absence of exercise (Iida et al., 2007). Whilst there is an increase in the central cardiovascular response during exercise, this returns to baseline acutely (5–10 min) post-exercise cessation.

The studies that have maintained pressure during rest intervals (continuous BFR) have generally found the externally applied pressure to increase HR, SbP, diastolic blood pressure (DbP) or double product (HR × SbP) compared with the same exercise in free flow conditions (Takano et al., 2005; Renzi et al., 2010; Kumagai et al., 2012; Vieira et al., 2013; Poton and Polito, 2015; Sugawara et al., 2015; May et al., 2017). Recent works have reported contrary evidence, potentially due to the fact that occlusion pressure was set relative to AOP (Neto et al., 2016; Libardi et al., 2017). Cardiac output seems not to be affected by BFR during exercise, as BFR groups proportionally increased HR and decreased SV compared to non-BFR groups (Takano et al., 2005; Renzi et al., 2010; Sugawara et al., 2015). The removal of the BFR cuff during rest intervals (intermittent BFR) appear to mitigate cardiovascular differences between BFR and non-BFR exercise (Neto et al., 2016). Studies removing the cuff between sets or between exercises have found no further variations in HR, (Rossow et al., 2011; Fahs et al., 2012; Downs et al., 2014; Neto et al., 2016), SbP or DbP (Rossow et al., 2011; Neto et al., 2016), CO or SV (Rossow et al., 2011; Downs et al., 2014) in the BFR group compared with the non-BFR group.

Changes in central hemodynamic response are lower following BFR-RE as compared to HL-RE (Rossow et al., 2011; Fahs et al., 2012; Downs et al., 2014; Brandner et al., 2015; Poton and Polito, 2015; Libardi et al., 2017), especially if the BFR stimulus is combined with AE (May et al., 2017). However, there is evidence that alterations to the peripheral flow BFR during light walking augments both peripheral (11%) and aortic (43%) systolic pressure compared to similar exercise without occlusion. Interestingly this effect appears to be centrally mediated as BFR exerts influence only on the outgoing, but not reflected, pressure waves (Sugawara et al., 2015). Of note, pressure handling affects the cardiovascular response to BFR-RE. Higher relative restrictive pressures induce higher cardiovascular responses to BFR-RE (Rossow et al., 2012) and may increase the potential risk associated with BFR-RE. Additionally, if pressure cuffs are not removed during rest intervals, BFR-RE could maintain blood pressure elevated as compared to HL-RE (Downs et al., 2014). On the other hand, BFR-RE results in greater post-exercise hypotension than HL-RE (Domingos and Polito, 2018).

### Peripheral Vascular Response to BFR-RE

BFR exercise has been shown to affect arterial compliance and endothelial function. Vascular compliance has most frequently been tested following BFR-RE (Credeur et al., 2010; Clark et al., 2011; Fahs et al., 2012; Hunt et al., 2012, 2013). In the short term, Fahs et al. (2012) found four sets of four different lower limbs exercises to affect both large and small artery compliance. BFR-RE increased large artery compliance to the same extent as LL-RE and HL-RE, whereas small artery compliance was more affected by HL-RE with no differences between LL-RE and BFR-RE groups. These data suggest a transient improvement of endothelial function following BFR-RE. However, this acute response does not seem to preclude long-term adaptations of vascular reactivity (Clark et al., 2011; Hunt et al., 2012, 2013) which may in contrast be negatively affected by chronic low intensity BFR-RE (Credeur et al., 2010). Other forms of application of the BFR stimulus have received less attention in the literature. BFR-AE has acutely shown to impair flow mediated dilatation (FMD) (Renzi et al., 2010), whilst others have reported BFR-AE to increase FMD in the long term (Iida et al., 2011).

Systemic vascular resistance (SVR) falls in muscle exercise due to vasodilation. The threat of the systemic pressure not meeting new regulatory set-point during exercise is compensated by an increased CO and sympathetic vasomotor tone. A mismatch between CO, sympathetic control of vasomotor system and local mechanisms of active hyperemia could result in hypotensive syncope (Hogan, 2009). Syncope episodes have not frequently been reported in BFR-RE literature (Nakajima et al., 2006), but they seem to be more frequent among practitioners and clinical settings where the threat is greater in any case (Patterson and Brandner, 2017). The application of BFR in the absence of any other stimulus increases SVR with a concomitant decrease of CO (Iida et al., 2007). SVR has shown to increase or to remain unchanged following BFR-RE (Takano et al., 2005; Rossow et al., 2011; Staunton et al., 2015; Libardi et al., 2017) or BFR-AE (Renzi et al., 2010; Staunton et al., 2015) and to be reduced following exercise (Fahs et al., 2012). Although the relationship between CO and SVR does not seem to represent a cardiovascular threat in BFR exercise, a steady CO coupled with an increased SVR could drive an increase in blood pressure, and adverse individual responses may not be discarded.

### Venous Thromboembolism

A thrombus is a solid mass of platelets, red blood cells, and fibrin mesh that typically forms as a response to vessel wall injury and is part of the normal healing cascade (Furie and Furie, 2008). Pooling of blood during episodes of stasis, which can happen during hospitalization or prolonged travel, can stimulate thrombus formation. A thrombus large enough to block blood flow, especially if located in the smaller vessels, can result in local tissue ischemia and subsequent tissue death. If dislodged it is termed an embolus and can result in a pulmonary embolism (PE) which can be life-threatening (Heit, 2015). Collectively, deep vein thrombus (DVT) and PE are termed venous thromboembolism (VTE).

Incidence rates of VTE have been estimated at 10 million cases annually (Raskob et al., 2014). Western Europe, North America, Australia and Southern Latin American yield consistent VTE results ranging from 0.75 to 2.69 per 1000 individuals per year. The incidence increases with age, 2–7 per 1000 in individuals >70 years old. The incidence is lower in Chinese and Korean ethnicity, however, the aging population may factor into an increasing VTE burden (Raskob et al., 2014). Several VTE risk factors have been identified and include medical conditions such as major orthopedic surgery, major general surgery, lower extremity paralysis due to spinal cord injury, pelvic, hip or long bone fractures, poly-trauma and cancer (Anderson and Spencer, 2003; Cionac Florescu et al., 2013). Additional risk factors include a history of prior VTE, obesity, immobility, oral contraceptives, family history of VTE, physical inactivity and genetic conditions that affect blood clotting (Anderson and Spencer, 2003). Pregnancy carries an elevated risk both in the perinatal and post-natal periods (Heit et al., 2005). It is much more common to develop a DVT in the lower extremities compared to the upper extremities, with approximately 10% of DVT formations being found in the upper extremities (Kucher, 2011). Finally, the insertion of central catheters during medical procedures make up the largest risk factor in upper extremities thrombus formations (Grant et al., 2012).

### BFR-RE and Venous Thromboembolism: Acute Measures

There is an inherent concern in the formation of a DVT due to the external compression on vasculature via an occlusive cuff during BFR-RE. Many of the published BFR-RE trials do not directly measure for VTE formation or use diagnostic imaging. However, the totality of the literature reveals minimal adverse events pertaining to VTE and clinically reported events have not been reported.

Most studies that have assessed for VTE after the application of BFR-RE have used direct blood markers for coagulation. Acute studies have not demonstrated a significant increase in blood coagulations via D-dimer and values, one of the most utilized clinical tests to rule out the presence of a DVT, after BFR-RE exercise (Nakajima et al., 2007; Fry et al., 2010; Madarame et al., 2010; Clark et al., 2011). Madarame et al. (2013) included prothrombin fragment (PTF) and thrombin-antithrombin III complex (TAT) testing to assess for increased thrombin generation immediately after training and found no significant increase. Additionally, C-reactive protein (CRP), a protein that has been linked to clot formation, was also assessed in one study and was not significantly elevated (Clark et al., 2011). Subjects performing BFR-RE at simulated elevation (8,000 ft) and at 6 degrees head down positioning did not demonstrate a significant rise in D-dimer, fibrin degradation product (FDP) or plasminogen activator inhibitor (PAI) (Nakajima et al., 2007). Only one study has assessed blood coagulation markers in a clinical population (Madarame et al., 2013). Nine subjects (7 men and 2 women) with a confirmed history of ischemic heart disease performed bilateral lower extremity knee extensions at 20% 1-RM with or without BFR. D-dimer, FDP and CRP were assessed before, immediately after and again 1 h after both exercise conditions. D-dimer and CRP was significantly elevated in both BFR-RE and free flow conditions, however, the values remained within a clinically normal range. Once adjusted for plasma volume (PV) the changes in each group’s values were no longer statistically elevated. CRP demonstrated the same non-clinically significant rise and after PV adjustment was not significant. FDP was not statistically elevated in either group.

Most of the acute studies have been performed on healthy populations [4 healthy (Nakajima et al., 2007; Fry et al., 2010; Madarame et al., 2010; Clark et al., 2011) vs. 1 clinical (Madarame et al., 2013)]. Furthermore, sex characteristics have trended toward male vs. female subjects (38 males and 4 females), and all have been performed on the lower extremities only (Nakajima et al., 2007; Fry et al., 2010; Madarame et al., 2010, 2013; Clark et al., 2011). All studies except for one, which compared BFR-RE at 30% 1RM to an 80% 1RM free flow control group, utilized LL-RE in both BFR-RE and free flow conditions (Clark et al., 2011). Standard pressures between 150 and 200 mmHg were used in all studies (Nakajima et al., 2007; Fry et al., 2010; Madarame et al., 2010, 2013) except for one study that used a pressure equal to 130% SBP (Clark et al., 2011). Future acute studies that focus on relative pressures, the upper extremities, clinical populations and female subjects are warranted.

### BFR-RE and Venous Thromboembolism: Chronic Measures

Most applications of BFR-RE in the clinical and research settings are done in a chronic manner over weeks and even months. Several papers have addressed VTE concerns in a chronic model. After 4 weeks of bilateral lower extremities exercise at 30% 1RM no changes in D-dimer, fibrinogen or CRP were noted (Clark et al., 2011). Similarly, 2 days a week for 12 weeks of BFR-RE at 20–30% 1RM did not significantly increase FDP, D-dimer or creatine kinase (CK) values in elderly subjects (ages 61–84 years; Yasuda et al., 2015a). The same authors found after 12 weeks of bilateral elbow extension and elbow flexion elastic band exercises no significant increase in D-dimer, FDP, or CK levels (Yasuda et al., 2015b). Chronic BFR-RE after knee surgery, 12 sessions over an average of 6 weeks, revealed no signs of thrombus formation as measured by duplex ultrasound scans (Tennent et al., 2017). A large epidemiologic questionnaire in Japan of over 12,000 subjects reported the incidence rate of venous thrombus at 0.055% and PE at 0.008%, of note a true medical diagnosis for PE was not confirmed (Nakajima et al., 2006). The value reported for DVT incidence in this study is lower than the reported in the general population in Asia (0.2–0.26%) which assumes a very low population risk (Klatsky et al., 2000).

The total time frame for chronic studies measuring VTE potential after BFR ranges from 4 to 12 weeks over four studies. There is much less gender bias in the chronic studies with a total of 35 men and 37 women tested. One study, utilized Doppler Ultrasound to personalize cuff pressure to 80% of AOP in the lower extremities and wide cuffs (11.5 cm; Tennent et al., 2017). The additional three studies used narrower cuffs on lower extremities (3–6 cm) and two used standard absolute pressure at an average of 196 ± 18 mmHg and the other utilized 130% of SbP (Clark et al., 2011; Yasuda et al., 2015a,b).

### BFR-RE and the Fibrinolytic System

Clotting in the vascular system after injury is part of the normal healing cascade and short periods of stasis can produce thrombus formation without adverse events. One mechanism to control the advancement of thrombus formation is through stimulation of the fibrinolytic system. Resistance training has demonstrated the ability to up-regulate the fibrinolytic pathway and has been demonstrated after just one exercise session and in healthy young participants and aged patients with coronary artery disease (CAD) (El-Sayed, 1993; de Jong et al., 2006). It would appear that BFR-RE stimulates the fibrinolytic system, as application of lower extremity BFR-RE increased tissue plasminogen activator (tPA, a thrombus-degrading protein in the epithelial cell) in healthy participants (Nakajima et al., 2007; Madarame et al., 2010). Additionally, the application of vascular occlusion without exercise has demonstrated a significant increase in fibrinolytic factors (Stegnar and Pentek, 1993; Nakajima et al., 2007). However, variables such as age, sex, and obesity may alter the fibronolytic response to exercise (Stegnar and Pentek, 1993).

### BFR-RE and at Risk Populations for VTE

Identifiable risk factors for VTE have been established and are a combination of endogenous characteristics such as obesity and genetic factors or exogenous triggering factors such as major surgery or pregnancy (Cushman, 2007). Advancing age is a non-modifiable risk factor for VTE formation. After the fourth decade of life, rates of incidence increase rapidly from 1 per 10,000 annually up to 5–6 per 1000 annually by age 80 (Silverstein et al., 1998). Studies addressing blood coagulation factors after BFR-RE, including D-dimer, FDP and CK, in elderly subjects have not demonstrated adverse effects. Three studies have included subjects with age ranges between 61 and 85 years and comprise both lower and upper extremities BFR-RE training (Fry et al., 2010; Yasuda et al., 2015a,b). One study has addressed BFR-RE in an aged clinical population (ischemic heart disease), and did not demonstrate an increase in blood coagulation factors (Madarame et al., 2013). Other risk factor group’s coagulation status after BFR-RE has not been directly studied. However, ongoing BFR-RE clinical trials in at risk populations (dialysis patients, femur fractures and joint arthroplasty) are ongoing (Takano et al., 2013; ClinicalTrials.gov, 2016; Clarkson et al., 2017b). Established clinical prediction rules to assess VTE probability in at risk subjects can be utilized prior to the application of BFR-RE to assist clinicians and researchers in appropriate candidates (Wells et al., 2000).

### BFR-RE and Reactive Oxygen Species

Oxidative stress can occur when the generation of reactive oxygen species (ROS) exceeds the ability of the antioxidant system to reduce the molecules (Garten et al., 2015). Deflation of a tourniquet cuff is associated with an increase in ROS and has been directly associated with ischemic reperfusion injuries after orthopedic surgery (Cheng et al., 2003). Additionally, resistance exercise can induce generation of ROS (Reid and Durham, 2002; Rodriguez et al., 2003; Nikolaidis et al., 2007). Moderate exposure to ROS is necessary to induce adaptive antioxidant defense mechanisms, however, chronic or high levels of exposure have been linked to disease conditions and signaling the blood coagulation system (Alfadda and Sallam, 2012; He et al., 2016).

Blood markers of oxidative stress include protein carbonyls, lipid peroxides and blood glutathione as well as antioxidants systems. The application of BFR-RE (20% 1RM) to bilateral lower extremities did not significantly raise lipid peroxide levels (Takarada et al., 2000a). When comparing BFR, in combination with LL-RE (30% of 1RM) and moderate resistance exercise (70% 1RM) only the BFR and moderate resistance groups demonstrated increases of protein carbonyls and blood glutathione (Goldfarb et al., 2008). Similarly, BFR alone increased oxidative stress but the addition of low-level exercise to BFR (30% 1RM) significantly attenuated protein carbonyls and glutathione status. Furthermore, one exercise bout or 1 week of high-frequency BFR-RE (1–2 sessions per day/3 weeks; 20–30%-1RM) does not appear to augment oxidative stress or antioxidant enzyme response (Nielsen et al., 2017a; Centner et al., 2018b). However, moderate intensity (70% 1RM) exercise with or without BFR both significantly elevated oxidative stress (Garten et al., 2015). Thus, overall the addition of BFR to LL-RE does not appear to increase oxidative stress or antioxidant defense, thus oxidative stress formation may be load, rather than BFR-dependent. Further work to understand the effect of BFR exercise on the oxidative stress responses, and thus the potential roll for this to act as a stimulus for adaptation, are required.

### Muscle Damage

Traditional HL-RE can induce muscle damage, particularly in those who are not experienced with exercise (Damas et al., 2016). This damage can be documented by direct and indirect markers and is most often associated with the eccentric phase of the exercise (Nosaka and Newton, 2002). The initial damage response is thought to occur due to overstretching of the sarcomere, resulting in z-line streaming as well as eventual disruption of the cytoskeletal matrix (Proske and Morgan, 2001). Muscle damage may also lead to activation of stretch-activated calcium channels or transient receptor potential channels which can increase intracellular calcium which can lead to destruction of sarcomeric proteins via calpain activation (Allen et al., 2005; Yeung et al., 2005). Following the initial damaging bout, there is often a secondary damage caused by the inflammatory response (Pizza et al., 2002). Given these effects, damage to the muscle can be determined directly via muscle biopsy, or it can be inferred indirectly through quantifying the symptoms thought to associate with a damaged muscle (Clarkson et al., 1986). These markers include a decrease in force production, decreased range of motion, muscle soreness, edema, and by measuring circulating levels of CK and/or myoglobin.

In extreme cases, exercise can be associated with a breakdown of striated skeletal muscle tissue, termed exertional rhabdomyolysis, that can lead to secondary pain, swelling and potential end organ damage (Tietze and Borchers, 2014). Cases of exertional rhabdomyolysis are typically associated with an exercise load that greatly exceeds the fitness and normal physical exertion of the participant, but have also been associated with high thermal loads, dehydration, or the use of certain medications (Zimmerman and Shen, 2013). It has been suggested that an exaggerated risk of rhabdomyolysis might occur as a result of BFR training, wherein metabolic stress is magnified despite the use of low-loads. Indeed, there are isolated case reports of rhabdomyolysis occurring through the use of BFR-RE (Iversen and Rstad, 2010; Clark and Manini, 2016; Tabata et al., 2016), however, analysis of the incidence rate from the published literature suggests the risk remains very low (0.07–0.2%) (Thompson et al., 2018). Survey data from Japan, where Kaatsu training has been practiced by a greater number of people, suggests a, similarly, low incidence of 0.008% (Nakajima et al., 2006). Thus, while exertional rhabdomyolysis during BFR exercise is possible, evidence does not currently suggest that the risk is inflated compared to traditional exercise.

A common concern of applying BFR with or without exercise is the possibility that this stimulus may lead to or even augment muscle damage through ischemic-reperfusion injury. Although ischemia-reperfusion injury is most commonly associated with long durations of severe ischemia (Blaisdell, 2002), it is possible that the combination of short duration BFR with muscle contraction could elevate the possibility of muscle damage with this type of exercise. The exercise-induced muscle damage response to BFR has been investigated in both the upper and lower body (Loenneke et al., 2014a). Muscle soreness, an indirect marker of muscle damage, is consistently elevated above baseline in the days following LL-RE in combination with BFR (Umbel et al., 2009; Wernbom et al., 2012; Thiebaud et al., 2013; Wilson et al., 2013; Sieljacks et al., 2016; Nielsen et al., 2017a). Large decreases in maximal torque production are often observed immediately post-exercise, however, the majority of studies suggest that torque returns back to or near baseline in the following days (Umbel et al., 2009; Wernbom et al., 2012; Thiebaud et al., 2013; Loenneke et al., 2014a). Muscle edema is consistently increased immediately post-exercise, but this edema decreases over time and is often back to baseline by 24–48 h (Thiebaud et al., 2013; Farup et al., 2015). Further, the few studies which looked at changes in range of motion found no differences across time (Thiebaud et al., 2013, 2014). While some studies have reported prolonged decrements in torque and prolonged edema, these changes are not usually different from a repetition matched control without BFR (Umbel et al., 2009; Wernbom et al., 2012) indicating that these changes are a result of LL-RE, not the application of BFR. Although CK and myoglobin are not often measured in the studies designed to assess the time course of muscle recovery, the majority of studies do not find a change in the days following exercise or training (Abe et al., 2006; Yasuda et al., 2015a; Nielsen et al., 2017a). It is noteworthy that a recent study did observe a more prolonged decrement in torque, edema, and increases in blood proteins (CK and myoglobin) following 5 sets of blood flow restricted exercise to volitional failure (Sieljacks et al., 2016). In line, applying a high frequent protocol (1–2 sessions/day) for 2 bouts of 5 consecutive exercise days interspersed by 10 days of rest recently showed an increase in CK levels during and a decline in torque production after the first 5 days of exercise (Bjornsen et al., 2018). Notably, when these protocols were followed by a second bout of exercise 10–14 days later, there were minimal changes from baseline suggesting that there may be a repeated bout effect with this type of training (Sieljacks et al., 2016; Bjornsen et al., 2018). However, two studies applying a strenuous high frequent protocol (1–2 sessions/day) for 10–15 exercise days have shown delayed (12–20 days post-intervention) muscle mechanical adaptations presumably as a result of prolonged myocellular stress (Nielsen et al., 2017a; Bjornsen et al., 2018). To our knowledge, only two studies have investigated damage directly at the fiber level and report that although there are signs of stress, there appeared to be no or only minor damage to the actual muscle (Cumming et al., 2014; Nielsen et al., 2017a).

In summary, the available evidence suggests that the application of BFR does not appear to induce a muscle damage response to LL-RE using single exercise protocols of up to 5 sets to volitional failure. We recognize that there may be individuals who are more susceptible to muscle damage than others, however, this would seem to be driven more by inherent differences in the individual than the application of BFR. Nevertheless, easing an individual into the exercise program while documenting indirect markers of muscle damage may help to better identify those who may be more susceptible to muscle damage and help the practitioner to mitigate risk not only to BFR but exercise in general. To this point, the one study that did note damage (Sieljacks et al., 2016), observed a robust attenuation with indirect markers of muscle damage in response to the next exercise bout. The majority of the studies investigating muscle damage have applied the same pressure to each individual so it is presently unknown what impact applying a relative pressure (e.g., percentage of limb occlusion) may have on this response. In addition, most studies have been designed to investigate the time course of muscle damage following a single bout of low load resistance exercise. Thus, little is known about how this time course would be impacted by additional bouts of resistance exercise within the same week, however, current evidence suggests that 1 or 3 weeks of strenuous high-frequent (1–2 sessions/week) BFR training does not induce apparent myocellular damage in recreational active individuals (Nielsen et al., 2017a). Lastly, although there is evidence that low intensity aerobic exercise in combination with BFR may be beneficial for augmenting muscular adaptations over time, it is presently unknown if there is a damage response to this mode of exercise.

## Conclusion

The aim of this article was to give an overview of the adaptations to different modes of BFR, methods of application and the safety considerations. The authors recommend the use of BFR combined with different forms of exercise (resisted, aerobic, passively), considering the volume and intensity, as well as the amount of cuff pressure, restriction time, size and cuff material. Tables 1–3 set out the parameters by which practitioners should use BFR based on up to date and current research in the area.

**Table 3:** Model of exercise prescription with P-BFR.

	Guidelines
Frequency	1–2 times per day (duration of bed rest/immobilization)
Restriction time	5 min intervals
Type	Small and large muscle groups (arms and legs/uni or bilateral)
Sets	3–5
Cuff	5 (small), 10 or 12 (medium), 17 or 18 (large)
Rest between sets Pressure	3–5 min Uncertain – higher pressure may be needed (70–100% AOP)
Restriction form	Continuous


## Author Contributions

All authors contributed to the writing and reading of the manuscript. All were involved in the design and agreed to the statements made by the review.

## Conflict of Interest Statement

The authors declare a potential conflict of interest. JO is affiliated with Owens Recovery Science. This is a private company who run educational courses on the topic area. The remaining authors declare that the research was conducted in the absence of any commercial or financial relationships that could be construed as a potential conflict of interest.
