# Corrigendum: Blood Flow Restriction Exercise: Considerations of Methodology, Application, and Safety

**DOI:** 10.3389/fphys.2019.01332

**Published:** 2019-10-22

**Authors:** Stephen D. Patterson, Luke Hughes, Stuart Warmington, Jamie Burr, Brendan R. Scott, Johnny Owens, Takashi Abe, Jakob L. Nielsen, Cleiton Augusto Libardi, Gilberto Laurentino, Gabriel Rodrigues Neto, Christopher Brandner, Juan Martin-Hernandez, Jeremy Loenneke

**Affiliations:** ^1^Faculty of Sport, Health and Applied Sciences, St Marys University, London, United Kingdom; ^2^School of Exercise and Nutrition Sciences, Institute for Physical Activity and Nutrition, Deakin University, Geelong, VIC, Australia; ^3^Department of Human Health and Nutritional Science, University of Guelph, Guelph, ON, Canada; ^4^Murdoch Applied Sports Science Laboratory, Discipline of Exercise Science, Murdoch University, Perth, WA, Australia; ^5^Owens Recovery Science, San Antonio, TX, United States; ^6^Department of Health, Exercise Science, and Recreation Management, Kevser Ermin Applied Physiology Laboratory, University of Mississippi, Oxford, MS, United States; ^7^Department of Sports Science and Clinical Biomechanics, University of Southern Denmark, Odense, Denmark; ^8^MUSCULAB – Laboratory of Neuromuscular Adaptations to Resistance Training, Federal University of São Carlos (UFSCar), São Carlos, Brazil; ^9^School of Physical Education and Sport, University of São Paulo, São Paulo, Brazil; ^10^Coordination of Physical Education/Professional Master's in Family Health, Nursing and Medical Schools, Nova Esperança (FAMENE/FACENE), João Pessoa, Brazil; ^11^Sport Science Department, Aspire Academy for Sports Excellence, Doha, Qatar; ^12^I+HeALTH Research Group, Department of Health Sciences, Faculty of Health Sciences, Miguel de Cervantes European University, Valladolid, Spain

**Keywords:** blood flow restriction exercise, kaatsu training, occlusion training, BFR exercise, resistance training

In the published article, there was an error regarding the affiliation for Johnny Owens. His affiliation is reported as Owens Recovery Science. We would like to add that this is a private company who run educational courses on the topic area.

A correction has therefore been made to the **Conflict of Interest Statement**:

“The authors declare a potential conflict of interest. JO is affiliated with Owens Recovery Science. This is a private company who run educational courses on the topic area. The remaining authors declare that the research was conducted in the absence of any commercial or financial relationships that could be construed as a potential conflict of interest.”

In addition to the above, after concerns were raised regarding the use of the phrase “Position Stand,” the original article has been updated accordingly. Also the word “Position Stand” must be removed from the article title.

The authors apologize for this error and state that this does not change the scientific conclusions of the article in any way. The original article has been updated.

